# A Deleted Deletion Site in a New Vector Strain and Exceptional Genomic Stability of Plaque-Purified Modified Vaccinia Ankara (MVA)

**DOI:** 10.1007/s12250-019-00176-3

**Published:** 2019-12-12

**Authors:** Ingo Jordan, Deborah Horn, Kristin Thiele, Lars Haag, Katharina Fiddeke, Volker Sandig

**Affiliations:** 1 ProBioGen AG, Herbert-Bayer-Straße 8, 13086 Berlin, Germany; 2 Present Address: Sartorius Stedim Cellca GmbH, Erwin-Rentschler-Str 21, 88471 Laupheim, Germany; 3 Vironova AB, Gävlegatan 22, 113 30 Stockholm, Sweden; 4 Present Address: Department of Laboratory Medicine, Karolinska Universitetsjukhuset i Huddinge, 14152 Huddinge, Sweden

**Keywords:** Modified vaccinia Ankara (MVA), MVA-CR19, CR.pIX, Suspension cell line, Vaccine production, Genome stability

## Abstract

Vectored vaccines based on highly attenuated modified vaccinia Ankara (MVA) are reported to be immunogenic, tolerant to pre-existing immunity, and able to accommodate and stably maintain very large transgenes. MVA is usually produced on primary chicken embryo fibroblasts, but production processes based on continuous cell lines emerge as increasingly robust and cost-effective alternatives. An isolate of a hitherto undescribed genotype was recovered by passage of a non-plaque-purified preparation of MVA in a continuous anatine suspension cell line (CR.pIX) in chemically defined medium. The novel isolate (MVA-CR19) replicated to higher infectious titers in the extracellular volume of suspension cultures and induced fewer syncytia in adherent cultures. We now extend previous studies with the investigation of the point mutations in structural genes of MVA-CR19 and describe an additional point mutation in a regulatory gene. We furthermore map and discuss an extensive rearrangement of the left telomer of MVA-CR19 that appears to have occurred by duplication of the right telomer. This event caused deletions and duplications of genes that may modulate immunologic properties of MVA-CR19 as a vaccine vector. Our characterizations also highlight the exceptional genetic stability of plaque-purified MVA: although the phenotype of MVA-CR19 appears to be advantageous for replication, we found that all genetic markers that differentiate wildtype and MVA-CR19 are stably maintained in passages of recombinant viruses based on either wildtype or MVA-CR.

**Electronic supplementary material:**

The online version of this article (10.1007/s12250-019-00176-3) contains supplementary material, which is available to authorized users.

## Introduction

Modified vaccinia Ankara (MVA) is a highly attenuated poxvirus investigated as vector for protective and therapeutic treatment also of immunocompromised human patients (McShane *et al*. 2002; Webster *et al*. 2005; Cebere *et al*. 2006; Milligan *et al*. 2016). It is not inhibited by pre-existing immunity and allows expression of large transgenes to elicit robust T cell responses (Sutter and Moss 1992; Sutter *et al*. 1994; Drillien *et al*. 2004; Liu *et al*. 2008).

MVA was derived from a vaccinia virus by repeated passage in primary chicken cells in 1954/55 (Mayr and Munz 1964). Productive replication is restricted to avian cells and very few mammalian cells such as BHK and cell lines derived from the Egyptian fruit bat (Carroll and Moss 1997; Drexler *et al*. 1998; Jordan *et al*. 2009a). The molecular basis for the narrow host range of MVA compared to the parental virus appears to be due to six deletions (deletion site I through VI, with the numerals increasing with the size of the deletion, not with position in the genome) and a number of more confined mutations (Meyer *et al*. 1991; Blanchard *et al*. 1998; Meisinger-Henschel *et al*. 2007).

We previously generated a continuous anatine suspension cell line (AGE1.CR.pIX) and developed chemically-defined media (Jordan *et al*. 2011) for a highly efficacious and safe vaccine production process for recombinant and wildtype MVA (Venkatraman *et al*. 2019; Folegatti *et al*. 2019). With this process, we also described the isolation of a novel genotype of MVA, termed MVA-CR, out of a non plaque-purified preparation (Jordan *et al*. 2013a). MVA has a linear dsDNA genome of 178 kb that contains approximately 177 genes in the core region and large inverted terminal repeats (ITRs). We recovered the sequence of a contiguous stretch of 135 kb of the core of the genomic DNA of an isolate at passage level 11. Within this region, three point mutations were observed. The mutations were nonsynonymous and localized to three structural proteins, A3, A9 and A34. A plaque-purified isolate of MVA-CR, called MVA-CR19, was further characterized and appeared to release a higher number of infectious units into the supernatant of infected cultures compared to those infected with wildtype. Because vaccinia virions are known to remain associated with the host cells (Blasco and Moss 1991; Blasco *et al*. 1993; Meiser *et al*. 2003; Husain *et al*. 2007), we hypothesized that the mutations contribute to a facilitated release of infectious units (Jordan *et al*. 2013a).

A high dose of 10^8^ infectious units are estimated to be required for vaccination because MVA is not amplified (replicated) at the injection site (Wyatt *et al*. 2004). Production of MVA poses certain challenges (Cottingham and Carroll 2013) that may be alleviated with a new strain such as MVA-CR that would allow simplified processes or even continuous production in true suspension cultures (Tapia *et al*. 2017; Vázquez-Ramírez *et al*. 2018). We have now characterized MVA-CR19 in greater detail and our results indicate that the genomic structure of this strain deviates from wildtype virus in more aspects than we had previously anticipated. Deletion site I near the left viral telomere is lost in what appears to be due to a recombination with the right telomere. We have also discovered one additional point mutation in a non-structural gene, MVA082L (L3L), and report here investigation of the contribution of the four CR-genotype mutations in the wildtype backbone.

Most importantly, from the perspective of using MVA-CR19 or parental MVA as a vector, all studied transgenes in deletion site III and all genetic markers are maintained stably without reversions, losses or rearrangements in plaque purified viruses for at least 20 passages.

## Materials and Methods

### Cell Culture and Viruses

CR.pIX cells from the muscovy duck (Jordan *et al*. 2009b) and the virus isolate MVA-CR19 (Jordan *et al*. 2013a) have been described previously.

CR.pIX cells were maintained in adherent format in DMEM:F12 medium supplemented with 5% bovine serum (γ-irradiated, Gibco 26140-079), or in suspension cultures in CD-U4 medium (GE Healthcare #G3321 or Biochrom #F9185) supplemented with 10 ng/mL LONG-R3IGF (Sigma, USA). Both media were also supplemented with 2 mmol/L GlutaMAX I (Life Technology, USA). Infection and propagation of MVA was performed in 1:1 mixtures of CD-U4 and CD-VP4 (Merck-Millipore #F9127) as described previously, usually with 2 × 10^6^ cells/mL, MOI of 0.01–0.1, and harvest 48 or 72 h post infection (p.i.) (Jordan *et al*. 2011). Suspension cultures were maintained in a shaking incubator (HT Multitron Cell, Infors AG, Switzerland) on a rotating platform with amplitude of 5 cm and rotation speed of 180 min^−1^. The CO_2_ atmosphere was set to 8% and temperature to 37 °C. All culture vessels, shake tubes (Tubespin 50, TPP Techno Plastic Products AG, Switzerland) or baffled shake flasks (Corning, USA), were equipped with 0.2 µm filtered lids to allow gas exchange. Suspension culture volumes were maintained at 20%–40% of the vessel size.

Infectious titers of MVA were determined in PFU/mL (plaque forming units) or FFU/mL (fluorescence forming units) as described previously (Jordan *et al*. 2013a) on Vero cells. Viruses were visualized in the non-permissive indicator cells by immunostaining or, where applicable, with help of the fluorescing reporters in deletion site III.

### Generation of Recombinant MVA

Recombinant MVA was generated by homologous recombination in adherent CR.pIX cells by adaptation of published methods (Kremer *et al*. 2012). Here, 1 × 10^6^ CR.pIX cells were seeded per well of a 6 well-plate. The culture monolayers were infected with receiving MVA with a MOI of 0.01 on the following day and transfected with 2.0 µg of shuttle plasmid for insertion into deletion site III. Point mutations were introduced by homologous recombination with a synthetic fragment that additionally contained silent diagnostic sites for restriction enzymes to confirm successful insertion and maintenance (Table 1). The recombination flanks ranged 600–1000 bp on each side.

**Table 1:** Summary of mutations observed in MVA-CR19 and silent mutations used as markers in recombinant viruses.

Gene	Mutation	Diagnostic site	Wildtype	Recombinant
MVA082L (L3L)	V110A	∆ *Hph* I (ggtga)	ttg gTg aga lys VAL arg	ttg gCg aga lys ALA arg
MVA113L		*Ava* I* (ctcgag) *Psp* XI* (vc/tcgagb)	tgT tcT TCt cys ser ser	tgC tcG AGt cys ser ser
MVA114L (A3L)		*Nco* I* (c/catgg)	tca atg gat ser met asp	tcc atg gat ser met asp
	H639Y		aga Cat att arg HIS ile	aga Tat att arg TYR ile
MVA120L (A9L)	K75E		aag Aag aat lys LYS asn	aag Gag aat lys GLU asn
		*Eco*R I* (g/aattc) ∆ *Xcm* I (ccan_9_tgg)	ccA aat tca ttt tgg pro asn ser phe trp	ccG aat tca ttt tgg pro asn ser phe trp
MVA121L (A10L)		K554K with *Sty* I (ccwwgg)	ccA aaG gtA pro lys val	ccC aaG gtC pro lys val
MVA145R (A34R)	D86Y	*Acc* I* (gt/atac) ∆ *Bsa*W I (a/ccgga)	aga ccg Gat act arg pro ASP thr	aga ccg Tat act arg pro TYR thr

The silent mutations in MVA113L and MVA121L are markers to confirm that recombination includes the complete flanks. Note that GenBank sequence U94848 lists a mutation (cca aGA gta, R554K) in A10L at this site. However, this deviation is corrected in a subsequent analysis so that U94848 and AY603355 are considered identical (Antoine *et al*. 2006).

Transfections were performed with Effectene Transfection Regent (Qiagen, Germany) according to the manufacturer’s instructions 90 min after infection, and the medium was replaced 24 h post transfection. The infected/transfected culture was harvested after 48 h to 72 h, sonicated, and used to infect cell monolayers in a 6-well plate. Plaques of the appropriate fluorescent phenotype were picked usually after another 24 h to 48 h and total DNA was isolated from aliquots of individual plaques using QuickExtract DNA Extraction Solution 1.0 (Epicentre, USA). Another round of plaque purification was initiated with the candidate recombinant virus preparations that passed the PCR analysis. The material for infection was obtained by sonication of cell harvests using a Vial Tweeter (set to 20 s of 100% cycle and 90% amplitude) that allows handling of closed sample caps to avoid cross-contamination (Hielscher, Germany). Viruses with parental genotype or incomplete recombination were not detectable within 3–8 rounds of plaque purification.

Virus passages to assay genomic stability was performed in CR.pIX suspension cultures in a volume of 5 mL with 1:1 mixtures of CD-U4 and CD-VP4. Cell density was 2 × 10^6^ cells/mL and MOI 0.01 (in blind passages a titer of 10^8^ PFU/mL was assumed in the previous passage). The infected culture was sonicated 48 or 72 h post infection to harvest virus.

### PCR Analysis of rMVA

80 µL of complete cell lysate was mixed with 20 µL of QuickExtract DNA Extraction Solution 1.0 (Epicentre, USA) and heated to 65 °C for 10 min and to 98 °C for 5 min. 4 µL of this preparation was subjected to PCR in a final volume of 25 µL with 0.15 µL Taq polymerase (Qiagen, Germany), 200 nmol/L each primer, and 125 µmol/L each nucleotide. The sequence of the primer pairs that span deletion sites I–VI of the viral genome were obtained from the literature (Kremer *et al*. 2012). The expected sizes of the amplification products are 291, 354, 447, 502, 603, and 702 bp for wildtype virus deletion sites I to VI (Kremer *et al*. 2012), and 1285 for deletion site III in MVA-CR19.GFP. Thermocycling was initiated with 94 °C for 80 s, followed by 35 cycles of 94 °C for 20 s, 55 °C for 20 s and 72 °C for 90 s, and terminated with 72 °C for 5 min. Amplicons were separated by electrophoreses in 1.5% agarose gels.

### Cloning of Shuttle Plasmids

The shuttle plasmid for deletion site III was cloned stepwise via insertion of the left and right flanks into pEGFP-N1 (Clontech, USA). The flanks were amplified from the genomic DNA of wildtype MVA with the primers shown in Supplementary Table S1. The left flank was cut with *Nhe* I (all restriction enzymes used in this study were obtained from New England Biolabs or Roche) and *Pci* I, the right flank with *Dra* III and *Not* I for sequential insertion into the same sites of pEGFP-N1 while maintaining the EGFP open reading frame. The artificial EL promoter (Chakrabarti *et al*. 1997) was generated by annealing two complementary 72 bp-oligonucleotides (TIP MolBiol, Germany) with the sequence ATC TGC TAG CAC GTG GAC TAG TAA AAA TTG AAA TTT TAT TTT TTT TTT TTG GAA TAT AAA TAA GAT CTT ACC on the conding strand. The annealing was performed after denaturation at 95 °C for 2 min followed by a ramp down to 56 °C with − 0.1 °C per second. This fragment was cut with *Bgl* II and *Nhe* I, precipitated with 300 mmol/L sodium acetate in two volumes of ethanol, purified by polyacrylamide gel electrophoresis, and inserted into the same sites of the pEGFP-N1 plasmid already containing the deletion site III flanks. Sequencing confirmed integrity of the shuttle plasmid but revealed a transition from ttG aaa ttt to ttA aaa ttt in the EL promoter that was not corrected and maintained as GFP expression was strong in rMVAs. A viral transcription terminator signal (T_5_NT, Yuen and Moss 1987) is contained in the right flank. The DsRed1 derivative mCherry was synthesized with codon-optimization for duck (Eurofins Genomic, Germany) and inserted in antisense orientation to GFP and under control of the late P11 promoter (Bertholet *et al*. 1985). The resulting dual expression cassette spans 1615 bp from EL to P11 promoter, the amplification product for deletion site III primers is 2087 bp long.

The shuttle plasmids for introduction of the point mutations D86Y in A34R and V110A in L3L into wildtype MVA were cloned only with fragments amplified out of MVA-CR19 genomic DNA. These mutations already contain by chance diagnostic restriction enzyme sites to confirm successful recombination (Table 1). The A34R shuttle plasmid was cloned by amplification of 1393 bp with primers shown in Supplementary Table S1. The primers contained additional restriction sites for *Nhe* I and *Xba* I, respectively, at the 5′ termini for insertion into pEGFP-N1 [out of dam^(−)^ bacteria] using these sites. The region of interest for the L3L shuttle plasmid was also obtained by PCR and inserted into pCR-Blunt II-Topo (“pTopo”) as described in the Zero Blunt TOPO PCR Cloning Kit (Invitrogen, USA).

The shuttle plasmids for the other point mutations, H639Y in A3L and K75E in A9L, were cloned by insertion of synthetic DNA (Eurofins Genomic) that contained the desired point mutation and silent diagnostic mutations (designed using http://resitefinder.appspot.com/).

For the generation of the shuttle plasmid for A3L a 2008 bp fragment of A3L was amplified (primer sequences shown in Supplementary Table S1) and inserted into pTopo. A 274-bp fragment therein from *Sac* I to *Swa* I was replaced with a synthetic DNA containing H639Y and the silent *Nco* I site. One flank of this shuttle plasmid had to be extended because first recombination attempts did not include the desired H639Y site: an additional synthetic DNA of 479 bp containing a new (but silent) *Ava* I site was appended to the *Nco* I-distal side using *Swa* I (in MVA) and *Spe* I (in pTopo).

Recombination of a 415 bp synthetic DNA that also contained a silent diagnostic mutation near to the desired K75E mutation transferred only the diagnostic mutation as well (revealed by sequencing of plaque-purified viruses). The flanks were therefore extended and additional diagnostic mutations were inserted so that K75E is framed by markers as follows: First, a 2905 bp fragment containing A9L and neighboring gene A10L was amplified out of wildtype MVA genomic DNA with primers shown in Supplementary Table S1. This 2905 bp fragment was cloned into pTopo to yield pTA10L. A synthetic DNA containing the diagnostic sites in A10L was then inserted via a three fragment ligation using *Pme* I (in the vector) to *Nsi* I (in the MVA insert) of pTA10L as new vector backbone, *Spe* I to *Nsi* I in the synthetic DNA to insert the silent *Sty* I diagnostic marker, and *Nsi* I to *Pme* I of the pTA10L to restore the initial amplification product. Three-fragment ligation to obtain pTLA10L-StyI was necessary to circumvent an additional *Spe* I site in the vector backbone. The A9L flank was inserted using a synthetic DNA fragment containing the K75E mutation framed by diagnostic silent mutations on both sides, *Eco*R I and *Bse*R I. This fragment was inserted via a three-fragment ligation to circumvent a *Tth111* I site in the vector, using *Tth111* I in A9L to *Eco*R V in the multiple cloning site of the synthetic DNA vector, *Tth111* I to *Pme* I in pTA10L and *Tth111* I to *Pme* I in pTA10L to restore the vector. The resulting shuttle plasmid contains a MVA-derived fragment of 3620 bp.

### Sequencing and RACE

Genomic DNA of plaque-purified MVA-CR19.GFP was isolated by polyethylene glycol precipitation out of 100 mL of infected CR.pIX cells at 2 × 10^6^ cells/mL as described previously (Jordan *et al*. 2013a). Sequences were obtained by GATC Biotech AG (Germany) with the PacBio RSII technology and assembled using an unforced (without guide sequence) algorithm.

Because large gaps at the left side of the genome remained after sequence assembly, and because PCR against the deletion sites indicated a loss of deletion site I (that is located near the left terminus of MVA) 5′-end RACE was performed. Primer D2 RII (Supplementary Table S1) against a 5′ terminal region still covered by the genomic sequence assembly was designed using the Clone Manager Professional suite version 9 (Sci-Ed Software, USA). This primer was extended on 500 ng of viral genomic DNA in 100 µL of 1 × PCR buffer, 1 × Q solution, and 5 U Taq and 0.2 U ProofStart Taq polymerase (all Qiagen, Germany), 0.4 µmol/L primer D2 RII and 0.05 mmol/L each dNTP. The thermocycler was programmed for 35 cylces of 94 °C for 10 s, 57 °C for 60 s, and 68 °C for 3 min (with 95 °C for 2 min at the start and 72 °C for 10 min at the end of the program). This PCR reaction was purified with the QIAquick PCR Purification Kit, 25 µL thereof were incubated with terminal transferase (TdT, New England Biolabs #M0315S) in a final volume of 50 µL of 1 × Tailing Buffer, 0.25 mmol/L CoCl_2_ and 0.1 mmol/L dCTP. The tailing reaction was preceded by denaturation at 94 °C for 3 min, followed by addition of 0.5 µL of the TdT and incubation at 37 °C for 30 min, and termination at 70 °C for 10 min.

A nested PCR was next performed to recover the 5′ extended and dC-tailed product using primers D2 and the universal anchored primer AAP in a final volume of 100 µL as described above for D2 RII primer extension but with an extension temperature of 59 °C for 60 s (instead of 57 °C).

This first nested PCR was diluted 1:50 and subjected to a second nested PCR in a final volume of 50 µL, without Q solution, primers GSPD2-R and AAP, with the same thermocycler program as in the first nested PCR. A fragment of approx. 700 bp was isolated and purified by agarose gel electrophoresis with the Qiagen Gel Extraction kit and sequenced with primers AAP and GSPD2-R. Primers were designed on this sequence as a diagnostic pair for amplification of 469 bp spanning the newly discovered recombination site (RS469).

The long-PCR for amplification of the presumed left ITR of MVA-CR19 was performed with primers D2 RII and ITR-M to obtain 21,312 bp on MVA-CR19 and 9360 bp on wildtype MVA (Fig. 3). The ITR-M primer binds in forward orientation from 533 to 553 and in reverse orientation from 165,956 to 165,976 in GenBank sequence AY603355 whereas primer D2 RII binds only once, in reverse orientation from 9869 to 9892. The possible amplicons are therefore 9360 bp and 165,444 bp (ITR-M single-primer amplification) with wildtype MVA as template, but not 21,312 bp. LongRange PCR (Qiagen) was performed in 50 µL final volume with 200 ng of viral genomic DNA according to the manual. The thermocycler program was initiated with 93 °C for 3 min; followed by 10 cycles of 93 °C for 10 s, 57 °C for 30 s and 68 °C for 15 min; followed by 25 cycles of 93 °C for 15 s, 57 °C for 30 s and 68 °C for 21 min with extension by 20 s per cycle. Restriction enzyme analysis with *Bcl *I, *Nru *I or *Apa*L I was performed with 3 µL of PCR product in 20 µL final volume and 0.5 µL of enzyme according to the manufacturer’s instructions.

### Electron Microscopy

Single-cell cultures (not suspended aggregates) of 20 mL × 2 × 10^6^ CR.pIX cells/mL were infected with MOI of 8 and cultivated for 30 h. The infected cells were collected by centrifugation for 5 min with 200 ×*g* and resuspended in 2.5% glutaraldehyde in 100 mmol/L phosphate buffer, pH 7.3, and stored at 4 °C until further processing. The fixed cells were rinsed in 100 mmol/L phosphate buffer, pH 7.4 and then treated with 2% osmium tetroxide in 100 mmol/L phosphate buffer, pH 7.4 at 4 °C for 2 h. They were next dehydrated in ethanol and acetone prior to embedding in LX-112 (Ladd, Burlington, Vermont, USA). Ultrathin sections (approximately 50-60 nm) were prepared using a Leica ultracut UCT (Leica, Wien, Austria) and contrasted with uranyl acetate followed by lead citrate. The ultrathin sections were examined in a Tecnai G2 Spirit BioTWIN transmission electron microscope (FEI Company, Eindhoven, The Netherlands) and digital images were acquired using a 2 k × 2 k side-mounted Veleta CCD camera (Olympus Soft Imaging Solutions, GmbH, Münster, Germany).

## Results

### Missing Deletion Site I

**Fig. 1:**
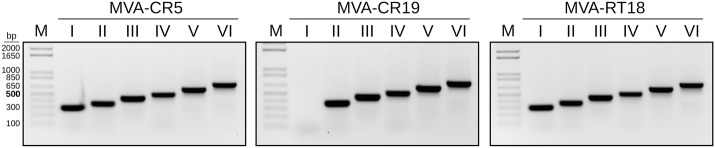
Deletion site I is missing in MVA-CR19 but not in wildtype MVA (passage 5 in CR cells, MVA-CR5) or in a MVA obtained by serial passaging on adherent cells of the Egyptian fruit bat (MVA-RT18 Jordan *et al*. 2013a). The expected sizes of the amplification products from deletion sites I through VI are 291, 354, 447, 502, 603, and 702 bp, respectively.

Recombinant MVAs can be characterized by a set of PCRs that are designed to amplify across each of the six deletion sites (Kremer *et al*. 2012). We used these PCR reactions to confirm insertion of the GFP-expression cassette into the commonly used deletion site III and were surprised that the expected signal for deletion site I was missing in MVA-CR19 derivatives. All other deletion sites gave amplicons of appropriate sizes in all tested viruses, and deletion site I signal was present in an earlier passage or an isolate passaged 18 times on the permissive fruit bat cell line (MVA-RT18; Jordan *et al*. 2013a) (Fig. 1).

**Fig. 2:**
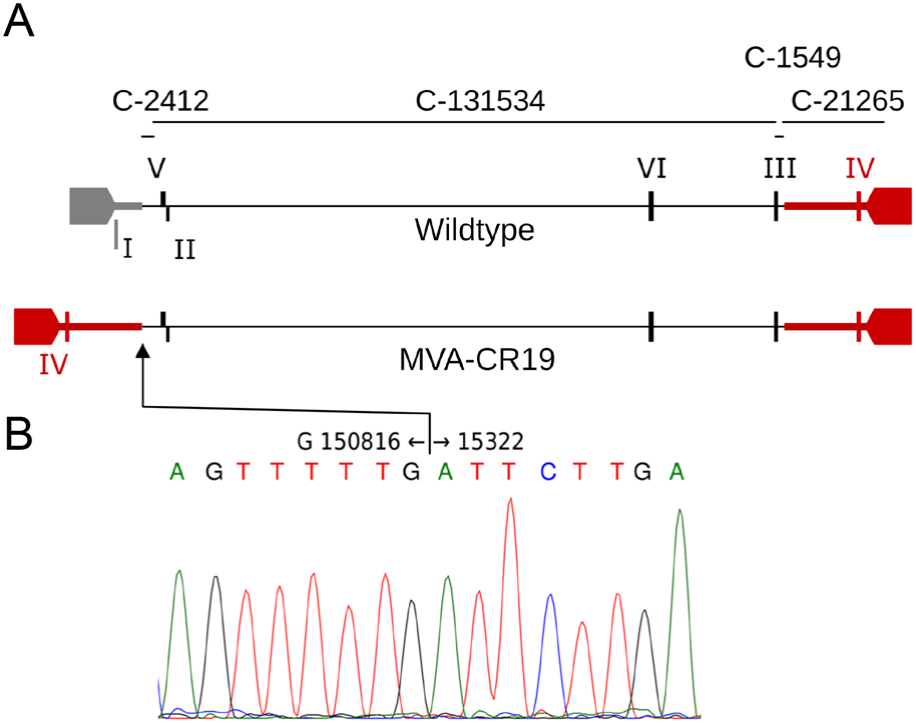
Proposed recombination in MVA-CR19. **A** Schematic of the wildtype genome with inverted terminal repeats (ITRs) and expected amplification products that span the six deletion sites (roman numerals). The bars preceeded with “C-” indicate the contigs that were obtained in previous (Jordan *et al*. 2013a) sequence analysis. The recombined region in addition to the ITRs is shown by the bolded line. **B** Sequence chromatogram of the recombination site, numbering with reference to GenBank U94848. The sequence of MVA-CR19.GFP has been submitted to Genbank with accession number KY633487.

The deletion site I is localized at the boundary of the left inverted terminal repeat and partially overlaps with the core region of the genomic DNA. The previously reported sequence of the genomic DNA of MVA-CR11, an ancestor of MVA-CR19, was obtained by unguided assembly and covered 135 kb of the genomic DNA that stretched downstream of deletion site I beyond deletion site III (Fig. 2A) (Jordan *et al*. 2013a). An additional sequence of 21 kb covering the right end of the genomic DNA was obtained by sequence assembly with GenBank entry U94848 as guide sequence and extended beyond deletion site IV into part of the ITR without any deviations from wildtype. Sequences from the left ITR were not recovered in the earlier experiments.

Absence of signals for deletion site I can also be observed in preparations of the ancestral (undeleted) chorioallantois vaccinia Ankara virus (CVA) (Suter *et al*. 2009). Although CVA has a broad host range and our earlier studies confirmed that MVA-CR19 cannot replicate in human HEK 293 or simian Vero cells (Jordan *et al*. 2013a), we could not exclude the presence of residual CVA-derived regions in our MVA isolate. We therefore attempted to detect the corresponding genomic origins of CVA by PCR. Because all reactions with primers that have been described to amplify CVA (Table 1 in (Suter *et al*. 2009)) were negative (data not shown), we suspected a loss of deletion site I due to genomic changes at the left end of the viral DNA of an authentic MVA.

To elucidate potential mechanisms for the changes we performed primer extension and TdT tailing, using the known sequence of the contig termed C-2412 (Fig. 2) as a starting point. The sequence of the PCR fragment obtained from the genomic DNA of MVA-CR19 reached from the core towards the left telomer but stopped at nucleotide 15,322 (using GenBank AY603355 as reference), and continued with the antisense strand towards the right telomer at nucleotide 150,816 (Fig. 2). A sequence as that obtained in Fig. 2B suggests that the left ITR of MVA-CR19 may have formed by recombination with the right ITR. The recombination site (RS) is downstream of deletion site I and upstream of deletion site IV.

**Fig. 3:**
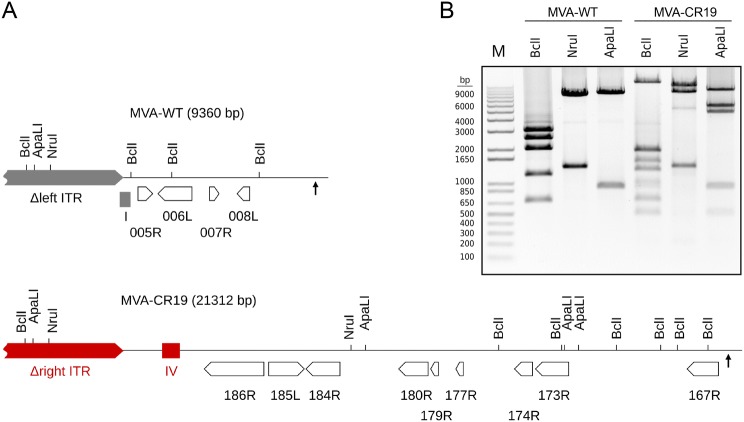
Confirmation of ITR rearrangement as shown in the previous figure. **A** Proposed structures of a PCR amplication products derived from the left ITR of the GenBank sequence U94848 and of MVA-CR19. Shown are genes (open arrows), deletion sites I and IV (filled boxes), ITRs (pointed grey and red rectangles), recombination site (up-pointing arrows) and target sites for restriction enzymes that were used to confirm the proposed structure of the left ITR. **B** Agarose gel electrophoresis of the long-PCR product shown in (**A**). Expected sizes for the 9360 bp amplicon of wildtype cut with *Bcl* I are 3007, 2518, 1992, 1172, and 671; with *Nru* I are 7998 and 1362; with *Apa*L I are 8464 and 896; for the 21,312 bp amplicon of MVA-CR19 cut with *Bcl* I are 13,634, 1890, 1498, 1269, 984, 886, 671 and 480; with *Nru* I are 11,257, 8693, and 1362; and with *Apa*L I are 9572, 5646, 4706, 896, and 492 bp.

To confirm that the proposed recombination has indeed occurred in MVA-CR19, we designed specific PCR reactions for the different genotypes. One primer binds distal in both ITRs and faces towards the center independent of whether it annealed to the left or right ITR. The other primer is complementary to a unique (non-repeated) sequence in the core of the genome and faces towards the left ITR. The expected amplification product with GenBank AY603355 as template is 9360 bp long and contains deletion site I. (An additional product of 165,444 bp can theoretically be obtained by a single-primer PCR initiating from the opposed viral termini.) A rearrangement of the right ITR to the position of the left ITR increases the amplification product from 9360 to 21,312 bp (Fig. 3A). Such different amplicons were indeed obtained for MVA-WT and MVA-CR19, respectively, and restriction fragment polymorphism with *Nru* I, *Bcl* I and *Apa*L I further demonstrated the expected identity of the obtained fragments (Fig. 3B).

The lost fragment in the left ITR contains MVA001L to MVA013L (with MVA014L as the first gene not affected by the deletion). Only MVA005R (C11R in vaccinia virus Copenhagen), MVA006L (C10L) and MVA008L (D7L) therein appear to be functional genes (Antoine *et al*. 1998; Meisinger-Henschel *et al*. 2007).

### Sequencing of MVA-CR19.GFP

We designed another guide sequence that contained the discovered recombination event in a sequence derived from Genbank entry AY603355. The new sequencing result was obtained with DNA isolated of a MVA-CR19.GFP preparation and covered a total of 145,636 bp (including the GFP expression cassette in deletion site III but again not successful in recovering the telomers) in three contigs of 15,557, 89,342 and 40,737 bp.

All previous mutations could be confirmed and only one additional point mutation was discovered, V110A in MVA082L (L3L in vaccinia virus Copenhagen). We also sequenced L3L directly by conventional chromatography method in preparations of passage 2 MVA, passage 11 MVA and the plaque-purified MVA-CR19, and observed two notable differences to the previous three point mutations: there appeared to be no visibly mixed population of L3L genotypes in MVA-CR11, and there were no indications of the presence of L3L in the passage 2 preparation [The A9L mutation of the CR-genotype was already visible in passage 2 of non-plaque purified MVA, and all CR genotype mutations were overlapping with wildtype sequence in chromatograms of MVA-CR11, see Fig. 2 of (Jordan *et al*. 2013a)].

### Fusion Phenotype

We previously observed a pronounced shift towards the novel strain in populations with mixtures of wildtype viruses and those that carry the MVA-CR mutations after repeated passage in suspension cultures (Jordan *et al*. 2013a). Such a shift may be caused if a viral genotype replicates faster, is associated with higher specific infectivity, or (as we hypothesize) reduces the affinity of its progeny viruses for the host cells. The property of MVA to remain associated with host cells is well characterized (Blasco and Moss 1991; Blasco *et al*. 1993; Meiser *et al*. 2003; Husain *et al*. 2007), with one consequence that syncytia can form if the viral fusion apparatus is activated in particles on the surface of a cell with contacts to neighboring cells (Ward 2005). We observed prominent syncytia formation by pH-mediated induction of the fusion apparatus only in cultures of CR.pIX cells infected with wildtype MVA but not with MVA-CR viruses [data not shown and (Jordan *et al*. 2013b)].

**Fig. 4:**
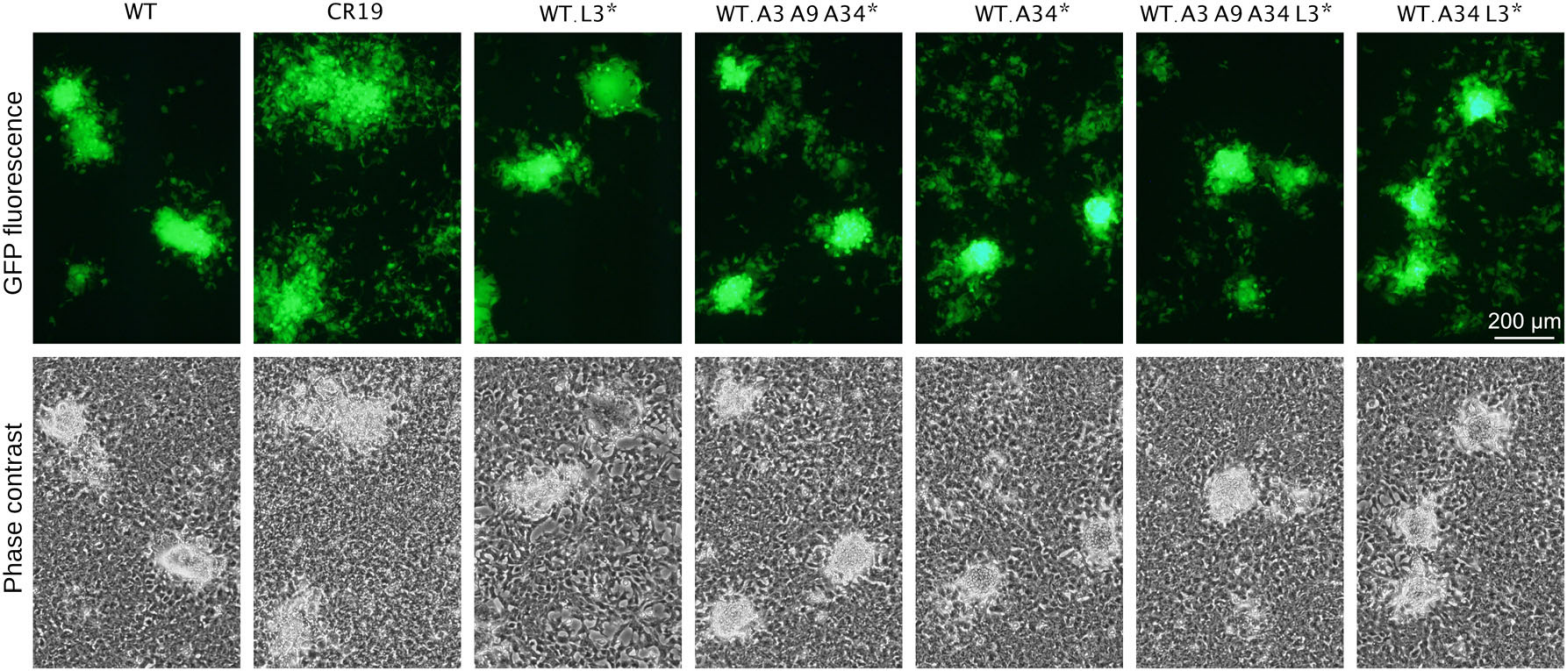
CR-associated mutations were inserted individually or in various combinations into the backbone of wildtype virus. GFP is expressed out of deletion site III to allow visualization of plaques without fixation and immunostaining. All infections were performed with MOI of 0.01 and plaque phenotype is shown 48 h p.i. WT, wildtype MVA that expresses GFP; WT.A34*, WT.L3* and WT.A3A9A34* denote WT viruses that express GFP and contain the respective CR-associated mutation; CR19 is MVA-CR19 that expresses GFP.

We introduced the point mutations of MVA-CR (Table 1) into the wildtype MVA backbone by homologous recombination of synthetic gene segments to investigate the contribution of the various mutations to the MVA-CR phenotype. These segments were designed to also contain silent mutations for diagnostic restriction enzyme polymorphism to confirm that plaque-purified recombinant viruses were of the intended genotype and without contaminating parental viruses. Insertion of a GFP reporter gene under control of a synthetic promoter into deletion site III facilitated study of plaque formation. Adherent monolayers of CR.pIX cells were infected with recombinant viruses to a MOI of 0.01 and fluorescence images taken at various time points (48 h post infection is shown in Fig. 4). The GFP signals from recombinant viruses that contained the A34R^CR^ mutation were scattered over large areas and the plaques exhibited only negligible spontaneous syncytia formation. Plaques formed by recombinant viruses with the A3L^CR^, A9L^CR^ and L3L^CR^ mutations resembled those formed by wildtype viruses.

We concluded from these experiments that viruses with the CR genotype have a decreased tendency to form spontaneous or pH-induced syncitia and that the mutation in A34 is the most important contributing factor for this phenotype. However, scattering of GFP signals compared to MVA-CR19 appeared to be not as extensive in wildtype viruses even with the D86Y mutation in A34.

### Virus Morphology by Electron Microscopy

**Fig. 5:**
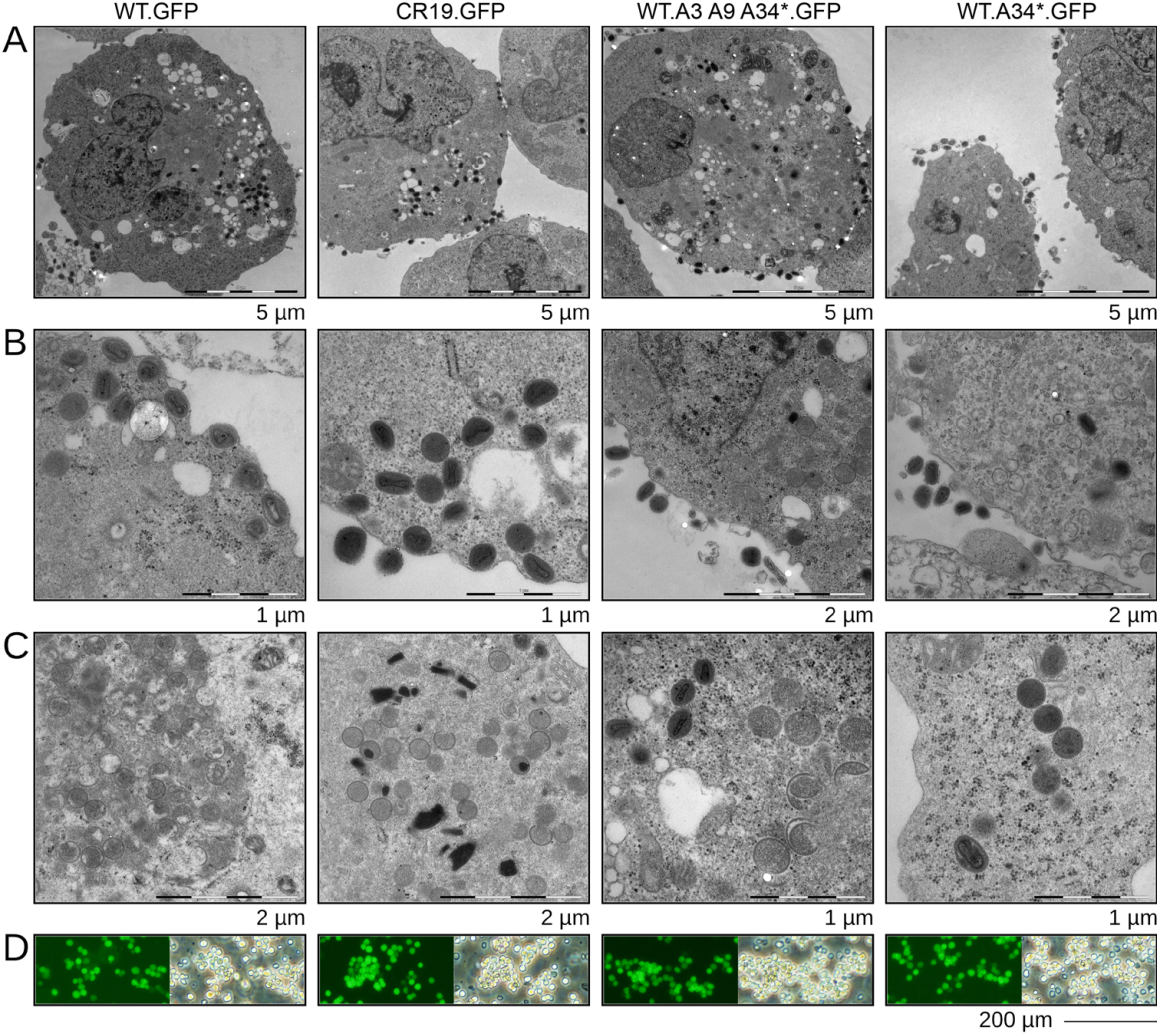
Electron microscopy images of recombinant MVAs. **A** Overview of infected cells with tendency of the non-wildtype viruses to be closer to the plasma membrane of the host cell. **B** There appear to be no differences in the morphology of wildtype and any of the recombinant viruses. A greater number of enveloped intracellular viruses were found in wildtype-infection and a greater number of intracellular mature viruses on MVA-CR19.GFP infected cultures. **C** Virus factories were very similar for all viruses with a slight tendency towards increased occurrence of DNA crystalloids (Grimley *et al*. 1970) in MVA-CR19.GFP infections. **D** Respective cultures infected with the GFP-expressing viruses prior to fixation for electron microscopy.

Vaccinia virus particles are complex and assembled in distinct steps (Gallego-Gómez *et al*. 2003; Roberts and Smith 2008). The mutations in MVA-CR19 affect three structural proteins in different layers of the virion (A3, A9 and A34), and one nonstructural protein within particles (L3) that may act as a cofactor for mRNA expression immediately following penetration into the host cell. We performed transmission electron microscopy 30 h post infection with GFP-expressing variations of wildtype and CR19 lineage to investigate whether the mutations impact properties of the various stages of MVA morphogenesis (Fig. 5).

Formation of the inner core of vaccinia virus particles initiates with sheets of lipids in a proteinaceous scaffold. These structures progress from open crescents into spheres that enclose a viroplasm of low electron density, the immature viruses (IVs). We did not observe differences in the early steps of morphogenesis (Fig. 5C).

Core wall formation and DNA condensation is one step towards intracellular infectious particles or mature viruses (MV). A3 contributes as a structural protein of the core and A9 may be involved to connect the core and MV envelope (Yeh *et al*. 2000; Heljasvaara *et al*. 2001; Byrd *et al*. 2002; Husain *et al*. 2006). There appeared to be no obvious structural differences in the transition of IVs to MVs of the various recombinant viruses in CR.pIX cells.

A fraction of the MVs migrate along microtubules towards the trans-Golgi network, where they are engulfed by cellular vesicles in such a way that the lumen of the vesicle is maintained. The resulting intracellular wrapped viruses (WVs) now have three lipid layers and are further transported to the cell periphery. If the outmost viral envelope fuses with the plasma membrane, a cell-associated virion with two membranes is formed. In this configuration, the virion can either infect neighboring cells (and occasionally induce syncitia formation) or detach to carry the infectious units as extracellular enveloped viruses (EVs) to distant sites. The outer membrane of the EV helps to protect the MV against the adaptive immune system of the host and must be shed prior to infection of new host cells (Moss 2012). The EV membrane is not very stable and often found to rupture spontaneously. However, detachment of EVs and disruption of the EV membrane can be further enhanced by mutations in the A33, A34 and B5 proteins (Blasco *et al*. 1993; Katz *et al*. 2002; Husain *et al*. 2007). EVs with partially or fully shed outer membranes are indeed a prominent occurrence especially in preparations of MVA-WT.A34CR.GFP-infected cultures (Fig. 5A, 5B). However, this result, although consistent with the expectation and previous experiments, should be interpreted carefully because the majority of EVs are lost during preparation for microscopy.

### No Observable Effects of the L3L Mutation

L3 is an essential protein for vaccinia virus replication (Upton *et al*. 2003). It is loosely incorporated into virions as a non-structural component and appears to enhance the infectious activity of the incoming particles by augmenting transcription or export of nascent mRNA (Resch and Moss 2005).

**Fig. 6:**
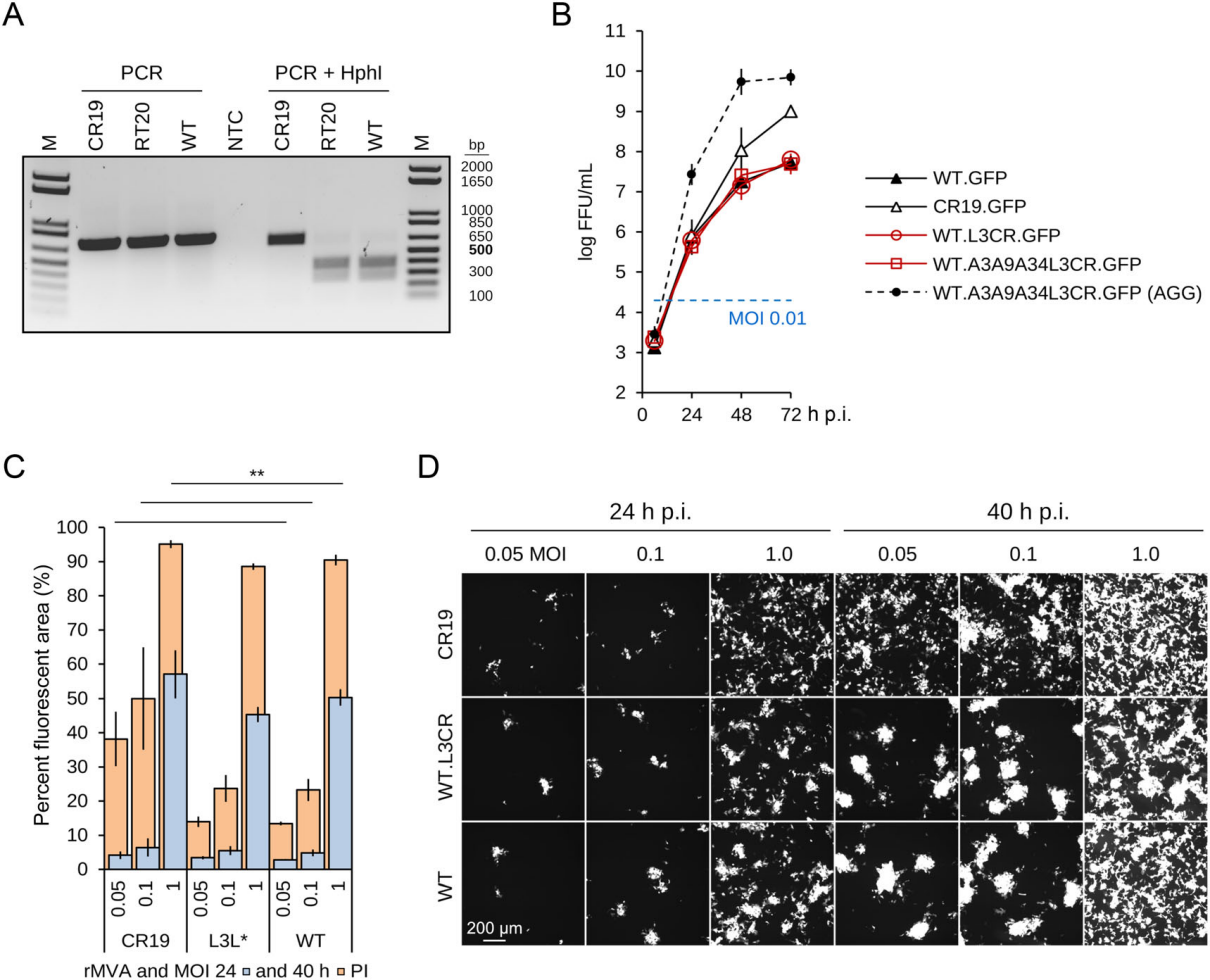
Characterization of the L3 mutation. **A** Restriction fragment length polymorphism due to the mutation in L3L in MVA-CR19. The 717-bp amplicon is expected to yield *Hph* I fragments of 649 and 68 bp for MVA-CR19 and 380, 269 and 68 bp for wildtype. RT20 denotes MVA after 20 passages in bat-derived R05T cells. **B**–**D** Two independent preparations of MVA-WT.L3LCR.GFP were used for these experiments. **B** Replication kinetic of the indicated recombinant viruses in single-cell suspension cultures. The dashed line shows replication of the indicated virus in the conventional process where aggregates are being induced. **C**, **D** Infection of adherent CR.pIX cells with GFP-expressing rMVAs. Depicted are mean and standard deviation of triplicates for MVA-WT.GFP and MVA-CR19.GFP, and of six values (two triplicates) of MVA-WT.L3CR.GFP. The log10 FFU/mL of the MVA preparations used for infection in this experiment were 8.5 (MVA-WT.GFP), 8.3 and 8.8 (MVA-WT.L3CR.GFP), and 9.8 (MVA-CR19.GFP). **Indicates significant differences between the corresponding 40 h time points of CR19 and wildtype recombinant viruses in a two-tail, independent *t*-test; the differences were not significant in a comparison between L3L-mutant and wildtype.

The newly discovered V110A point mutation leads to a restriction site polymorphism (an *Hph* I site is deleted) that allowed a comparison of MVA-CR19 and MVA-CR19.GFP to sequences obtained from wildtype virus or viruses passaged on the fruit bat cell line. The *Hph* I site polymorphism complements sequencing and was detectable in wildtype and bat-cell passaged MVA viruses (MVA-RT20) but not in MVA-CR19 (Fig. 6A).

Insertion of the V110A mutation into wildtype virus did not result in obvious changes to the plaque phenotype 48 h p.i. (Fig. 4). The L3 mutation also appeared not to affect infectious titers, and viruses with that mutation in the wildtype backbone replicated without differences to the wildtype reference in single-cell suspensions (Fig. 6B). MVA-CR19.GFP replicated to the expected high titers also without induction of aggregates in the same experiment, and a wildtype virus with all of the observed mutations of the CR genotype replicated to very high titers only if cell aggregates were induced.

We also tested for enhanced infectivity by comparing the expansion of foci in adherent cells infected with different recombinant viruses. A NyONE cell imager (SynenTec GmbH, Germany) was used to quantify how much of the cell area is covered with GFP-expressing (infected) cells at two time points (24 and 40 h p.i.) and increasing MOI (0.05, 0.1 and 1). Because correct MOI is an important parameter in this study, two separate preparations of MVA-WT.L3LCR.GFP (the L3L mutation of MVA-CR19 in the backbone of wildtype virus) were tested. As shown in Fig. 6C, 6D, there appeared to be no differences in the spread of viruses in the first 24 h and at a MOI below 1. Only MVA-CR19.GFP appeared to have a slight initial advantage at MOI of 1. At 40 h post infection, there were again no significant differences in the GFP-positive area between wildtype and the L3L mutant, independent of MOI, whereas MVA-CR19.GFP occupied a significantly larger area of the infected cell monolayer (for example at MOI 0.05, 38% for MVA-CR19 versus 13% for the viruses with the wildtype background).

### Stability of the Different Virus Species

**Fig. 7:**
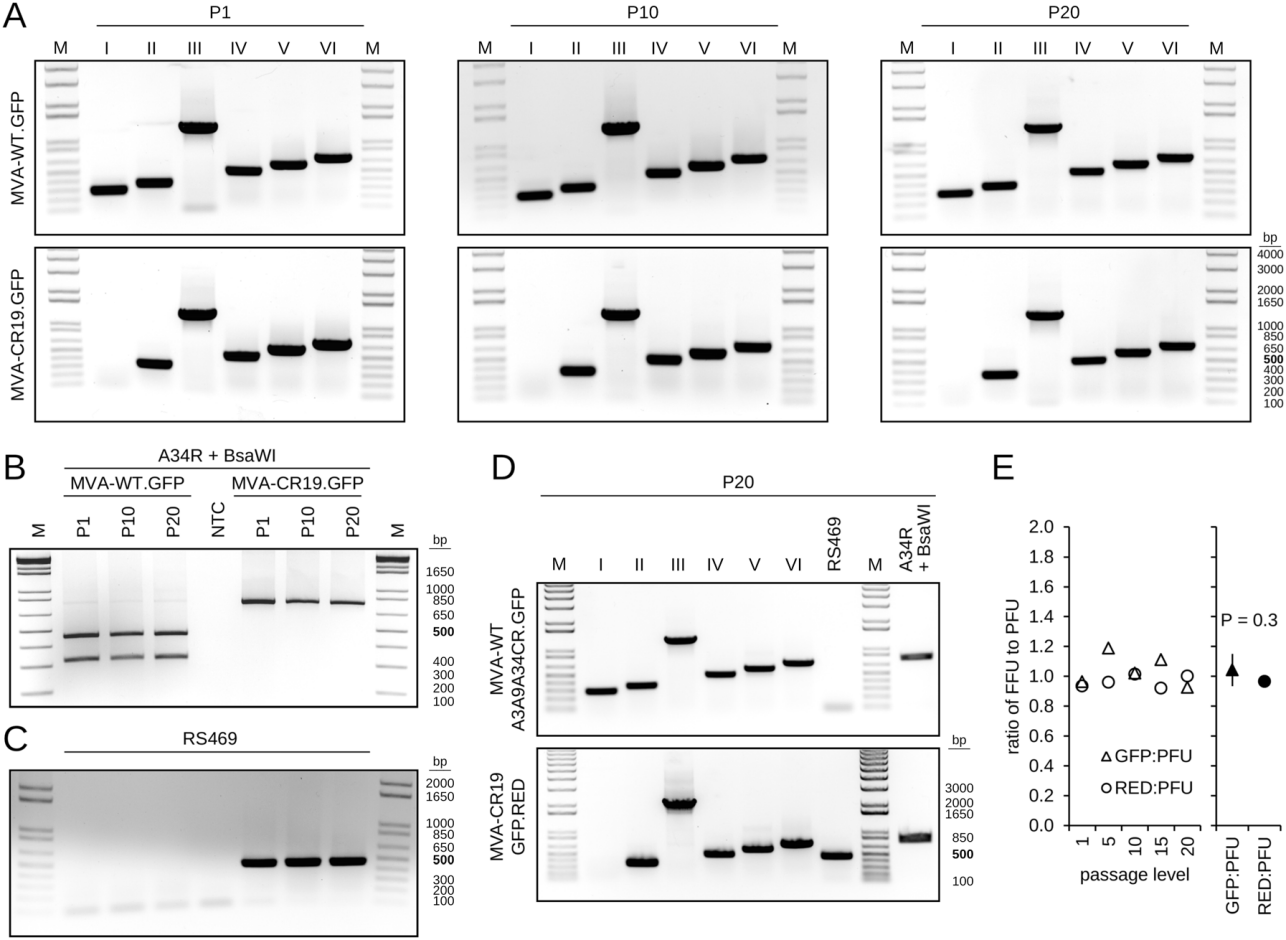
Stability of MVA genotypes in CR.pIX cultures. **A** Plaque-purified recombinant wildtype and CR19 isolates that contain a GFP-expression cassette in deletion site III were passaged 20 times in CR.pIX suspension cultures. Note absence of deletion site I signal in CR19 derivatives and the expected shift in size of the deletion site III amplification product due to the GFP cassette (from 447 to 1285 bp). **B** The viral DNA that was purified in **A** also exhibits a stable pattern of the restriction fragment length polymorphism (RFLP) in the A34R gene for both isolates over the passaging interval. **C** The presence of the RS469 amplification product that replaces deletion site I in CR19 derivatives is found in passages of CR19 but not in those of the wildtype. The samples were applied in the same sequence as in **B**. **D** Stable patterns of the deletion sites and the RFLP in recombinant viruses that carry the point mutations of the CR genotype in a wildtype backbone or a GFP and mCherry (RED) dual expression cassette in the CR19 derivative. **E** Infectious titers obtained with MVA-CR19.GFP.RED dual expression vector were determined at the indicated passage levels by determination of PFUs with immunostaining against vaccinia virus proteins, or via determination of the green or red fluorescence signals. The ratios of the infectious units is shown in the chart. A decrease of FFU relative to PFU would indicate loss of the expression cassettes. Titrations based on GFP tend to be higher than those based on mCherry in dual-expression constructs because some excitation of mCherry also occured under GFP fluorescence conditions. The mean of all ratios is 1.00 ± 0.09, there is no significant difference between the mean values of the ratios of GFP-FFU:PFU and mCherry-FFU:PFU at a 99% confidence level (paired *t*-test).

Maintenance of transgene expression, replication properties and degree of attenuation depend on the genomic stability of viral vectors. Although virus isolates with deletions and rearrangements in the ITR (Moss *et al*. 1981; Pickup *et al*. 1982; Paez *et al*. 1985; Qin *et al*. 2011) and transient gene amplification in response to selective pressures by the innate immune system (Elde *et al*. 2012) have been described previously, MVA is also known for high genetic stability (Antoine *et al*. 2006). We next investigated stability of genetic markers of wildtype MVA and MVA-CR19 by propagation in the duck-derived continuous suspension cell line in chemically-defined medium (a substantial deviation from the chicken-derived primary adherent cultures in the presence of serum that were used in generation of MVA). Plaque purified recombinant viruses that contained GFP or the dual GFP and mCherry expression cassette in deletion site III were passaged 20 times in the CR.pIX suspension cultures (Fig. 7). Background of the viruses were either wildtype or MVA-CR, or a wildtype virus with point mutations of the CR genotype in the affected structural genes. We observed no loss of deletion site I in the wildtype virus (Fig. 7A), no conversions in A34R (neither towards the CR genotype nor towards wildtype, Fig. 7B), no changes at the recombination site in MVA-CR (Fig. 7C), and no fluctuations in the expression of the two reporter genes that could indicate rearrangements in deletion site III (Fig. 7E).

## Discussion

A novel genotype of MVA has been isolated previously out of a non-plaque purified preparation of MVA (Jordan *et al*. 2013a). Certain mutations that characterize this strain appear to improve propagation of the virus in suspension cultures and may facilitate production processes. We here extend the analysis to further characterize what we consider to be a new, hitherto undescribed and highly stable vector configuration.

The point mutation D86Y in A34R of MVA-CR19 was associated with profound changes in the plaque phenotype and appears to be mainly responsible for the improved mobility of viruses in infected cultures. Although the D86Y mutation has not been reported previously to our knowledge, the involvement of A34 in release of cell-associated viruses from the host cell has been demonstrated in the literature (Blasco *et al*. 1993; Husain *et al*. 2007).

The point mutation in A9L of strain MVA-CR can be detected in chromatograms already in passage 2 preparations of non-plaque purified MVA, and the combination of the point mutations in A3, A9 and A34 of strain MVA-CR visibly replace the wildtype sequence starting with passage 11 (Jordan *et al*. 2013a). We were therefore surprised that a combination of all single amino acid substitutions in a wildtype backbone did not enhance replication to levels of MVA-CR19. A3 and A9 are structural proteins of the viral core (Heljasvaara *et al*. 2001; Kato *et al*. 2004) and the crescent-derived membrane of the virions (Yeh *et al*. 2000), respectively, and appear to be involved in early steps of morphogenesis towards intracellular mature virions. Mutations in structural proteins of the core may augment genome mobilisation during penetration, or may improve the transition from immature to mature particles. It is one limitation of our study that we could not perform the elaborate experiments that elucidate very early events of the infectious cycle (Cyrklaff *et al*. 2007). However, plaque morphology, replication kinetic and electron microscopy of productively infected cells did not reveal qualitative differences between wildtype and various mutant viruses based on the wildtype backbone.

In addition to the point mutations, MVA-CR19 differs profoundly from the wildtype at the left terminus. It appears that a large replacement of the left ITR by the right ITR lead to the loss of three and duplication of at least nine functional genes (Fig. 3). Attempts to sequence the telomers failed, but if we combine the Genbank sequence U94848 and the result of the diagnostic long-PCR, then the left genomic region that characterizes MVA-CR19 may have expanded from 15,327 bp to 27,108 bp. The rearrangement may therefore have increased the size of complementarity between the two telomers but has not affected the GC content (16.3 vs. 16.6 and 17.7 vs. 17.5%, for CR19 vs. wildtype left telomers).

Heterogeneity and rearrangements in the ITRs of vaccinia viruses has been described previously (Moss *et al*. 1981; Pickup *et al*. 1982; Paez *et al*. 1985; Qin *et al*. 2011). The ITRs of poxviruses, similar to the multigene families in the genome of the distantly related Asfariviruses (Portugal *et al*. 2015), contain partially redundant sets of immunomodulatory factors and determinants for host range (Antoine *et al*. 1998; Meisinger-Henschel *et al*. 2007; Dobson and Tscharke 2015). The rearrangement of the ITR can therefore impact properties of vaccinia virus vectors:

Deletion of MVA005R (C11R in vaccinia virus Copenhagen) may interfere with the capacity of MVA to engage extracellular signal-regulated kinase 2 (ERK2) that causes NF-κB activation (Martin *et al*. 2012). While vaccinia viruses interfere with NF-κB activation (Oie and Pickup 2001; Shisler and Jin 2004; Mohamed and McFadden 2009), MVA is reported to have lost the defensive factors against NF-κB pathways (Antoine *et al*. 1998). The effect on replication of viruses is difficult to predict since NF-κB activation furcates into signaling pathways that lead to antiviral responses and inflammation (Santoro *et al*. 2003). Tolerability and transgene expression may be improved by loss of MVA005R if activation of NF-κB pathways is indeed less vigorous.

Deletion of MVA006L (C10L) in the left ITR may be compensated by the duplication of MVA184R (B16R) in the right ITR. Both proteins are antagonists for the proinflammatory IL-1β. However, because the two factors act by different mechanisms (Alcamí and Smith 1992; Kluczyk *et al*. 2002), the replacement of one by the other in MVA-CR19 still may have consequences. The presence of a viral IL-1β decoy was shown to reduce virulence and febrile reactions to infection (Blanchard *et al*. 1998), and deletion of the immune evasion factor was shown to enhance Th1-mediated immunogenicity in a vaccinia virus mouse model (Staib *et al*. 2005).

Deletion of MVA008L (D7L) has also been proposed previously to both increase reactogenicity and the safety profile of MVA (Smith *et al*. 2000; Falivene *et al*. 2012). The D7 protein is secreted by the infected host cell and interferes with the function of interleukin-18, a central signal molecule for antiviral responses by the innate and adaptive immune systems.

The B1 kinase may have increased in activity. It is encoded by MVA167R, the first gene that is duplicated by the ITR recombination. The B1 kinase is recruited to the viral factories and appears to interfere with an innate defense of the host cell against cytoplasmic replication of DNA (Wiebe and Traktman 2007; Olson *et al*. 2017). The effects of B1 appear to be adjusted by a non-essential factor (Olson *et al*. 2019) that is also duplicated by the ITR recombination, the B12 pseudokinase (MVA 180R).

B5R (MVA 173R) is also duplicated by the rearrangement. B5 is a structural protein involved in the intracellular wrapping with Golgi-derived membranes that lead to formation of extracellular virions (Engelstad and Smith 1993). Interestingly, this factor has recently been shown to interact with A34 (Monticelli *et al*. 2019), the protein with the apparently most active point mutation in MVA-CR19. Whether the mutation in A34 impacts the interaction with B5, and effects that this may have, will be the focus of future studies.

Another gene that is duplicated and where increased gene dosis may explain advantages for the replication of MVA-CR19 is MVA186R. This gene encodes 68k-ank, the only protein with an ankyrin repeat motif (ANK) and F-box remaining in MVA. Viral ANK/F-box proteins can connect the cellular ubiquitin ligase with selected target proteins and are implicated in the regulation of host range and defense against innate immune responses (Herbert *et al*. 2015). One model posits that the 68k-ank protein may tag poxviral capsid components in the viral factories for ubiquitination (Sperling *et al*. 2008; Mercer *et al*. 2012; Liu *et al*. 2018). These components would be protected from degradation during particle assembly but would be recognizable by the proteasome immediately after infection and uncoating (Mercer *et al*. 2012). Degradation of the viral core by the cellular machinery would thus promote the release of the viral genome for the next step in the infectious cycle. Poxviral particles contain more than 3% molar abundance of ubiquitin (Chung *et al*. 2006). If ubiquitination by 68k-ank is important for genome mobilisation after infection, and if activity of 68k-ank is limiting, then a gene doubling may be beneficial for replication of MVA-CR19.

We differ from some of the previous studies in that we here observe an extensive rearrangement at the termini that appears to improve [rather than interfere with (Dobson and Tscharke 2015)] the replication of the affected virus. At least one of the point mutations and especially the rearrangement appears to be associated with improved replication of MVA-CR19. However, even with a replicative advantage we observed no changes in any of the investigated markers for at least 20 passages. Our study therefore confirms the high genetic stability of plaque-purified MVA vectors. The recombination between the left and right parts of the genome of MVA-CR19 caused deletion of C11R, C10L, and D7L, and doubling of B1R, B5R B12R and 68kD-ank. Preliminary experiments in non-permissive human and porcine cell lines suggest an improved expression of transgenes with MVA-CR19 compared to wildtype virus without affecting the host range. Future studies will examine immunologic properties of MVA-CR19 *in vivo* in suitable animal models.

## Electronic supplementary material

Below is the link to the electronic supplementary material.

Supplementary material 1 (PDF 61 kb)
