# Supplementary material for: A Deleted Deletion Site in a New Vector Strain and Exceptional Genomic Stability of Plaque-Purified Modified Vaccinia Ankara (MVA)
Source: Virol Sin. 2019 Dec 12;35(2):212–26. doi: 10.1007/s12250-019-00176-3 (PMC7198643; doi:10.1007/s12250-019-00176-3)
Supplement: Supplementary file 1 — Supplementary material 1 (PDF 61 kb) [file 12250_2019_176_MOESM1_ESM.pdf]

VIROLOGICA SINICA

Electronic Supplementary Material

A Deleted Deletion Site in a New Vector Strain and Exceptional Genomic Stability of Plaque-Purified Modified Vaccinia Ankara (MVA)

Ingo Jordan[1]✉, Deborah Horn[1], Kristin Thiele[1,2], Lars Haag[3,4], Katharina Fiddeke[1], Volker Sandig[1]

1. ProBioGen AG, Herbert-Bayer-Straße 8, Berlin 13086, Germany
2. Present Address: Sartorius Stedim Cellca GmbH, Erwin-Rentschler-Str 21, 88471 Laupheim, Germany
3. Vironova AB, Gävlegatan 22, 113 30 Stockholm, Sweden
4. Present Address: Department of Laboratory Medicine, Karolinska Universitetsjukhuset i Huddinge, 14152 Huddinge, Sweden

Supporting information to DOI: 10.1007/s12250 -019-00176-3

Table S1. Primers used for cloning of shuttle plasmids and for RACE.

Primer	Primer sequence	Expected size
Deletion site III left flank f × r	AGG ACA TGT-TTG GTG GTC GCC ATG GAT GGT × TAC CGC TAG C-T ACC AGC CAC CGA AAG AG	928 bp
Deletion site III right flank f × r	TGG GCG GCC GC- TTT GGA AAG TTT TAT AGG × TGG CAC GTA GTG - CCG GAG TCT CGT CTG TTG	699 bp
A34R shuttle sequence f × r	AAT GCT AGC-GCG GAA TCA TCA ACA CTA CCC × GCT CTA G-ATT GTT CCC GCA ACT ACG GTC	1393 bp
L3L shuttle sequence f × r	CTC TAC GGG CTA TTG TCT C × TGA ATA CCC GTA CCG ATG	717 bp
A3L shuttle sequence f × r	GCA GAA GAA CAC CGC TTA GG × ATG GAA GCC GTG GTC AAT AG	2008 bp
A9L/A10L shuttle sequence f × r	TTG AAA TAG CGC CAG TCC TCC × ACT ACG GCG GCA TTA TGT TCTC	2905 bp
RS469 f × r	ACG GTC CTG TAG TAT CTG × CGG CAT GTG GAG TGT CTT TAT C	469 bp
ITR long-PCR D2 RII × ITR-M	GGT GTA TAG AGT TCA CAG TAG × CTT GCA CAT GTC TCC GAT ACG	(*)
D2 RII	GGC GGC ATG TGG AGT GTC TTT ATC	
D2	GGT GTA TAG AGT TCA CAG TAG	
AAP	GCC ACG CGT CGA CTA GTA CGG GII GGG IIG GGI IG, with I for inosine	
GSPD2-R	GGA GGT GGC TCT CGA TGA AC	

All sequences are listed from 5' to 3'; f × r for forward and reverse primers. D2 RII, D2, AAP and GSPD2-R are primers for RACE. (*) Expected sizes for the ITR long-PCR are 21312 (MVA-CR19), 9360 (wildtype) and (theoretically) 165444 bp for an ITR-M single-primer PCR.
